# Tectonic–surface carbon-cycle dynamics: toward resolving cross-scale coupling challenges

**DOI:** 10.1093/nsr/nwaf433

**Published:** 2025-10-13

**Authors:** Liang Zhao, Gang Lu, Jianfeng Yang, Xinxin Wang, Zhengtang Guo

**Affiliations:** Key Laboratory of Deep Petroleum Intelligent Exploration and Development, Institute of Geology and Geophysics, Chinese Academy of Sciences, China; Marine Science and Technology College, Zhejiang Ocean University, China; State Key Laboratory of Lithospheric and Environmental Evolution, Institute of Geology and Geophysics, Chinese Academy of Sciences, China; State Key Laboratory of Lithospheric and Environmental Evolution, Institute of Geology and Geophysics, Chinese Academy of Sciences, China; State Key Laboratory of Lithospheric and Environmental Evolution, Institute of Geology and Geophysics, Chinese Academy of Sciences, China

## Abstract

This paper proposes through a numerical example that two-phase flow modeling provides a novel approach to address the challenge in modeling the cross-scale coupling between the tectonic and surface carbon cycles despite extreme viscosity contrasts, which unravels how deep Earth tectonics regulate atmospheric composition over geological time.

Surface carbon is transported into the mantle via subducted slabs and is subsequently returned to the atmosphere through carbonated melting and volcanic degassing [1,2], together constituting a tectonic–surface carbon cycle. The scientific community has gradually recognized that the release and absorption of CO_2_ caused by deep Earth tectonic activities regulate the composition of Earth’s atmosphere over long timescales (≥10^6^ years), thereby influencing climate evolution. However, one of the most fundamental questions in geosystem studies—the mechanisms of interaction, especially the cross-scale coupling (CSC) between the tectonic and surface spheres—remains poorly understood.

To comprehend the CSC effects between tectonic processes and atmospheric *p*CO₂ over geological time, previous numerical modeling studies have put forward several theories, all of which are still hotly contested. Release-dominated, absorption-dominated or mixing-effect-dominated are the three groups into which Zhao *et al.* [3] categorized the models that were previously in use. Although each of the three prominent mechanisms explains some findings, none can fully account for all observations, pointing to an inaccurate depiction of the controlling factors. The mechanism that controls CSC is still a grand challenge because it is too difficult to determine causal relationships in covarying signals of the tectonic and the surface changes due to their different timescales. The relatively weak fluctuations caused by long-term tectonic events are often overprinted by stronger short-term surface changes. Only when tectonic fluctuations continuously exceed a certain threshold in the long term can they be recorded in geological signals. We refer to these extreme fluctuations as tipping points in covarying tectonic–surface systems. Therefore, it is reasonable to reframe the broader challenge—understanding the mechanisms driving CSC—into an observable and simulatable topic: what mechanisms are responsible for initiating and rebalancing these extreme episodic events? This research approach also holds referential significance for studying other complex systems with CSC effects, such as the driving mechanisms behind volcanism, earthquakes and even the geodynamo.

To evaluate causal relationships in covarying signals, one common approach involves selecting typical episodic tectonic–surface events for forward modeling and comparing the simulation results with observational data, thereby establishing predictive models. Within this coupled framework, scientists have turned to the carbon cycle—which offers a relative abundance of data—as an ideal platform to integrate interior and surface processes and investigate tectonic–surface interactions. Moreover, the pathway of the tectonic carbon cycle is relatively well constrained [3]: (i) tectonic degassing releases carbon through four major pathways: volcanism at mid-ocean ridges, subduction zones and hotspots, as well as diffuse degassing along continental faults and fractures; (ii) in contrast, the largest carbon flux from the surface into Earth’s interior occurs via silicate weathering, followed by CO₂ sequestration through marine carbonate burial. Notably, plate subduction is the only tectonic process capable of transporting significant amounts of CO₂ beyond the lithosphere into the mantle.

Over 40 years ago, two generations of simulation methods of the tectonic carbon cycle were proposed. At the beginning, the tectonic carbon cycle was simplified as a mathematical representation in box models such as GEOCARB [4], which relied on mass balance among carbon reservoirs (boxes) and isotopic constraints. This kind of zero-dimensional approach is simple, efficient and provides first-order insights, but it has obvious limitations. First, it adopts homogeneity assumptions, ignoring spatial heterogeneity within different reservoirs (e.g. chemical heterogeneity in the mantle), leading to large uncertainties in flux estimates. For instance, the estimated fate of carbon in subduction zones may have errors exceeding ±50%. Second, it does not account for time-dependent effects within the system, causing internal changes to immediately reflect in the system’s output, making it difficult to capture dynamic adjustment processes. It therefore neglects CSC between subsystems, resulting in an oversimplified continuous change that fails to accurately characterize the tipping points for transition events. Accurate numerical methods are crucial for understanding the dynamics of the carbon cycle across Earth’s interior and surface. To overcome these limitations, a fundamental step is to accurately simulate the phase transitions and chemical reactions of carbon-bearing materials along their entire pathways. This enables the correct characterization of time-varying flux functions for carbon-bearing materials traversing deep and surface spheres, ultimately allowing reconstruction of the initiation and rebalancing processes of extreme covarying events.

The second-generation methods are represented by 2D or 3D modeling approaches applied to the tectonic–surface carbon cycle that incorporate the transport behavior of H_2_O and CO_2_ under varying temperature–pressure conditions, enabling preliminary investigations into the general patterns of deep decarbonization and carbon convergence [5,6]. These methods provide an effective tool for simulating the tectonic carbon cycle over timescales of ∼10–30 Myr. Furthermore, Liu *et al.* [7] presented the first application of a spatially continuous model—integrating climate and silicate weathering (representing the tectonic carbon cycle)—on a geological timescale by using a simplified boundary-condition-coupled approach. This simplified coupling approach treats the interaction between tectonic and atmospheric spheres as episodic (∼ Myr) rather than continuous. As a result, it does not capture the impact of the atmospheric system on the tectonic system.

Addressing CSC challenges requires acknowledging observational evidence: the migration of carbon/H_2_O-bearing minerals during deep tectonic activity is a complex process. It involves multicomponent and multiphase flow interactions that operate across multiple timescales. When rheological contrasts between solid and fluid phases become significant, accurately characterizing the migration processes of these carbon/water-bearing minerals presents a formidable technical challenge. Traditional single-phase modeling approaches based on Stokes equations typically require viscosity contrasts to remain below 6–7 orders of magnitude to maintain computational accuracy [8]. Yet, while mantle viscosity estimates range from 10^18^ to 10^20^ Pa s or higher, carbonated magma as a fluid phase exhibits viscosities as low as ∼1 Pa s, creating viscosity contrasts that far exceed the capacity of conventional single-phase geodynamic models. This necessitates the incorporation of additional physical processes for the fluid phase, such as Darcy’s law for describing fluid flow through porous media. A commonly employed solution involves implementing a simplified Darcy flow driven by pressure gradients under constant permeability conditions. However, this approach yields inconsistent and inaccurate fluid velocity predictions because it neglects several critical fluid–solid interaction mechanisms: (i) carbon-bearing fluid migration-induced porosity changes that dynamically modify permeability structures; (ii) bulk viscosity variations caused by porosity changes that govern compaction efficiency; (iii) fluid pressure gradient adjustments resulting from fluid migration that subsequently regulate migration rates; and (iv) fluid pressure changes that may react back on the solid matrix, potentially triggering the deformation of solid earth. The coupling between the solid and fluid phases thus gives rise to additional dynamic behaviors, such as solitary waves, that are absent in single-phase systems. This emergent phenomenon exemplifies a hallmark of complex systems, in which the collective behavior of the whole exceeds the sum of its individual parts.

We propose that the solid–fluid interaction mechanism is the key to addressing CSC-related challenges because the solitary wave is exactly the result of the interactive force between two coupled systems. One important tectonic event in the carbon cycle is the periodic characteristic of mantle plume tracks (Fig. 1a). In the classical view, a mantle plume consists of a plume head and a continuous conduit that feeds magma into the head. Interaction between the moving plate and the nearly fixed plume tail leaves a chain of volcanic tracks on the surface. Observations show that plume tracks are not continuous, but typically separated as calderas, which is not compatible with the predicted continuous plume tail from single-phase modeling. To resolve this discrepancy, we applied a novel method of a coupled two-phase flow and the new result exhibits periodic porosity waves with pulses of melt supply (Fig. 1b). Furthermore, our calculations demonstrate a linear relationship between the logarithms of the interval period (or distance) and the shear viscosity of the solid matrix, which can be expressed as: $lo{g}_{10}t = mlo{g}_{10}( \eta )$. This relationship implies that the observed periodicity is governed by the interaction between the fluid flow and the solid matrix. We propose that the non-linear dependency of the permeability on the porosity causes melt to accumulate in local porosity maxima and drains melt from the region surrounding them, producing obstructions that excite the formation of additional porosity waves. This series of experiments reproduce the CSC effects associated with the triggering and restoration of equilibrium during lithosphere–plume interactions.

**Figure 1.**
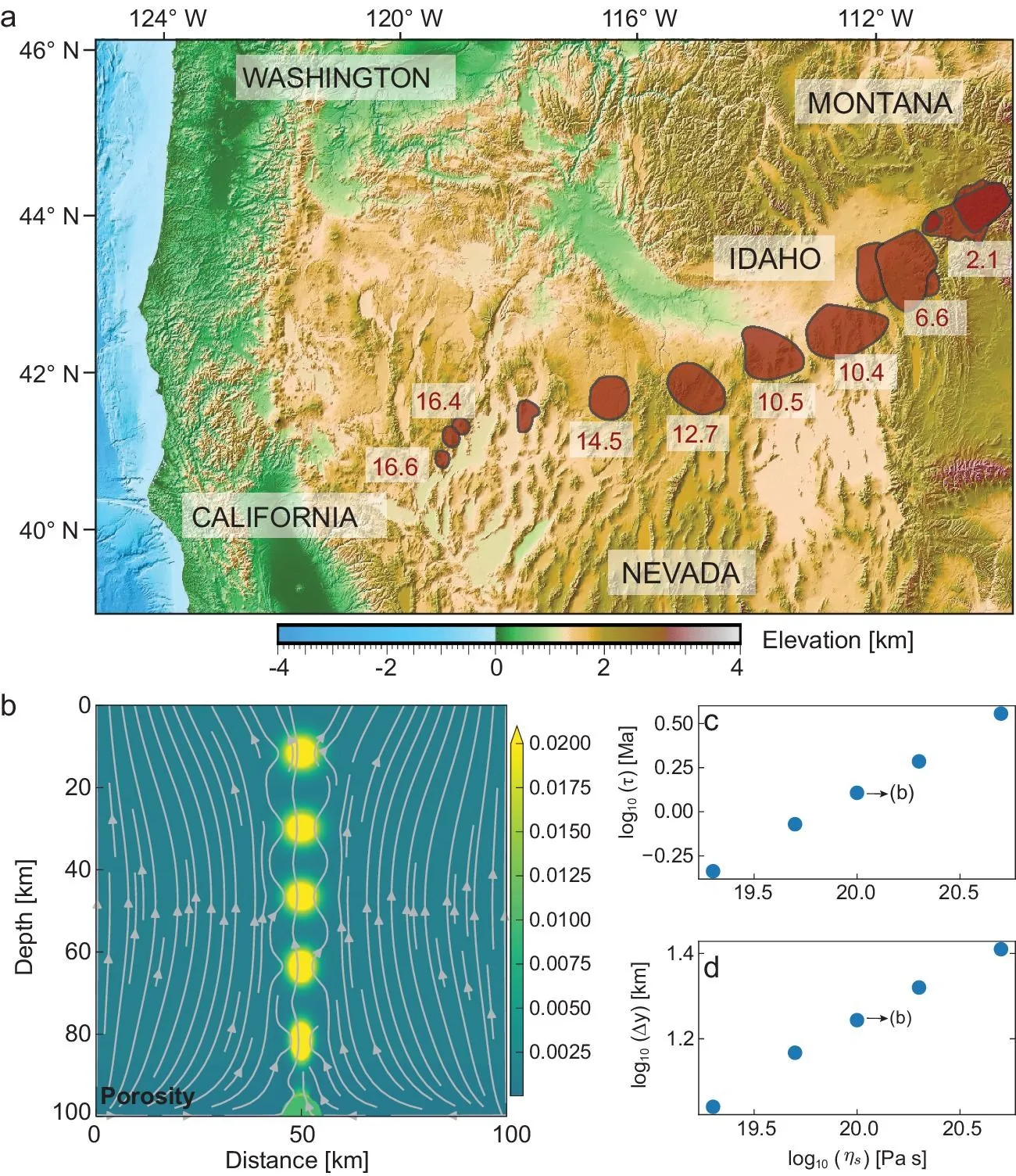
An example showing the cross-scale activity of mantle plume tracks related to a two-phase flow. (a) Map of Yellowstone hotspot track, with separated hotspot calderas indicating cyclic magma eruption. Numbers in white boxes show ages (unit: Ma) of calderas. Modified from Ref. [9]. (b) Two-phase flow model (porosity waves) for plume rising in this study. Shown are porosity fields overlain with fluid streamlines (gray lines). The model has an initial background porosity of *ϕ * = 10^–3^, except in the semicircle domain in the bottom, where the porosity is 10 times higher and remains constant through time. The solid matrix has a linear viscosity of *η*_s_ = 10^20^ Pa s and a fluid viscosity of *η*_f_ = 1 Pa s, with subscripts s and f indicating the solid and fluid phases, respectively. The density of the solid matrix is 3000 kg/m^3^, while the fluid phase is 500 kg/m^3^ lighter than the solid phase. Permeability depends on porosity in cubic law as $k = {k}_0{\phi }^3$, where ${k}_0$ = 5 *×* 10^–9^ m^2^ is the reference permeability. The top boundary has a free surface, with a constant fluid pressure boundary condition *P*_f_ = 0, implying that fluid is allowed to escape from the model surface. All other boundaries are free slip for solid velocity and impermeable for fluid flow. Porosity-dependent bulk viscosity is used, which takes the form of *ξ * = $\frac{{{\eta }_{\mathrm{s}}}}{\phi }$. (c) Interval period and (d) interval distance of porosity wave as a function of the shear viscosity of the solid matrix. The parameters used in (b) are labeled.

We envision that the next generation of tectonic–surface process simulators will need to address the coupling between two Navier–Stokes fluid systems operating at tectonic and surface timescales: when cumulative changes in the longer-period tectonic system exceed a critical threshold, both tectonic–surface systems undergo simultaneous adjustments to reach a new equilibrium state. Accurately describing these periodic, tectonically triggered CO₂ outburst events is a complex task. It necessitates the application of two-phase flow modeling approaches. Currently, our understanding of the carbon cycle primarily derives from geochemical analyses of abundant surface rock samples. While these data offer invaluable insights into magmatic dynamics, they predominantly reflect grain-scale equilibrium between solid and fluid phases, as fluids migrate orders of magnitude faster than do solids along the grain boundaries. The lack of precise fluid migration histories poses significant challenges in capturing the full spectrum of interactions between fluid flow, chemical transport and reaction processes [10]. Two-phase flow modeling presents a transformative approach to bridge this knowledge gap. By precisely resolving fluid migration velocities and pathways, this methodology enables the integration of physical properties across multiple scales.

Precise two-phase flow modeling coupled with detailed thermodynamic

calculations represents a cutting-edge research frontier in carbon-cycle physics. Although the plume-formation example presented here spans a rheological contrast of 20 orders of magnitude (Fig. 1), reflecting the current state of the art, this range remains relatively small compared with the solid earth–atmospheric rheological differences inherent in tectonic–surface coupling. Further technical advancements will be required to adequately address such extreme ranges in future work. Nevertheless, from a mathematical perspective, we have identified a novel approach that enables new investigations toward resolving CSC challenges.
